# Supplementary material for: Bottom-Up Strategy to Forecast the Drug Location and Release Kinetics in Antitumoral Electrospun Drug Delivery Systems
Source: Int J Mol Sci. 2023 Jan 12;24(2):1507. doi: 10.3390/ijms24021507 (PMC9861055; doi:10.3390/ijms24021507)
Supplement: Supplementary file 1 [file ijms-24-01507-s001.zip › ijms-2167163-supplementary.pdf]

Supplementary Materials

^{1}H-NMR of the proligand

N, N′ dimethyl-4-[(E)-dimethylaminodiazenyl]-5-carboxamide imidazolium iodide

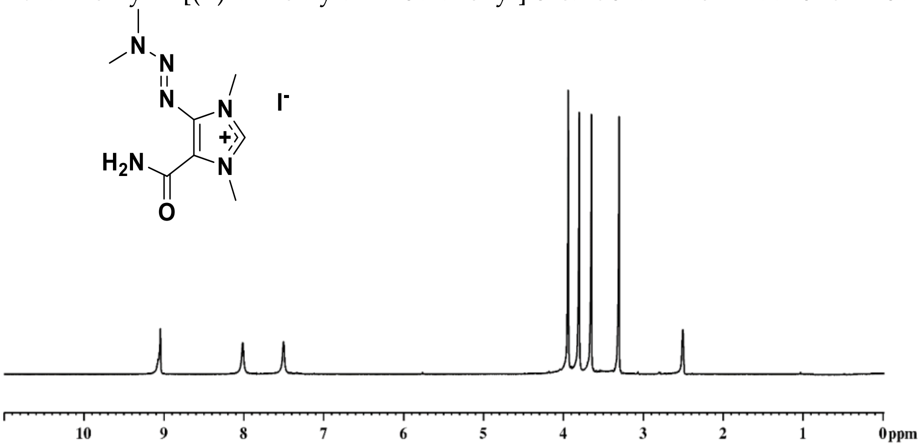

Figure S1. **^{1}H-NMR** (400 MHz, DMSO-d_6) ppm δ: 9.0_9 (s, 1H, NC**H**N), 8.0_1, 7.5_0 (s, 2H, N**H**$_2$), 3.9_4 (s, 3H, NC**H**$_3$), 3.8_0 (s, 3H, NC**H**$_3$), 3.6_5 , 3.4_0 (s, 6H, N(C**H**$_3$)$_2$).

^{13}C-NMR of the proligand

N, N′ dimethyl-4-[(E)-dimethylaminodiazenyl]-5-carboxamide imidazolium iodide

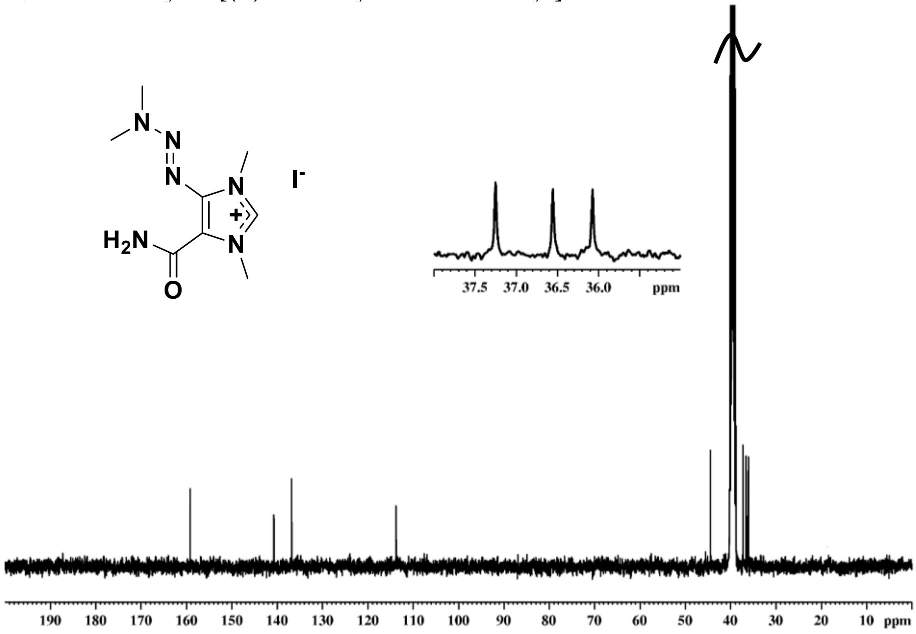

Figure S2. **^{13}C-NMR** (100 MHz, DMSO-d_6**)** ppm δ: 159.1_1(***C***ONH$_2$), 140.6_8 (C=***C***(N=N-N)), 136.7_8 (N***C***HN), 113.7_4 (C=***C***(CONH$_2$), 44.4_2 (NC***H***$_3$), 37.2_5 (NC***H***$_3$), 36.5_5, 36.0_7 (N=N-N(**C**H$_3$)$_2$).

N, N′ dimethyl-4-[(E)-dimethylaminodiazenyl]-5-carboxamide imidazolium iodide

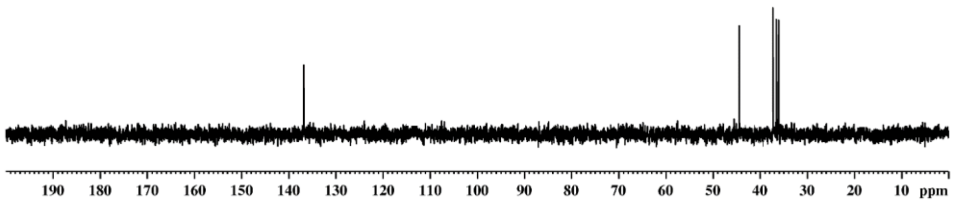

Figure S3. **DEPT 135 of the proligand**

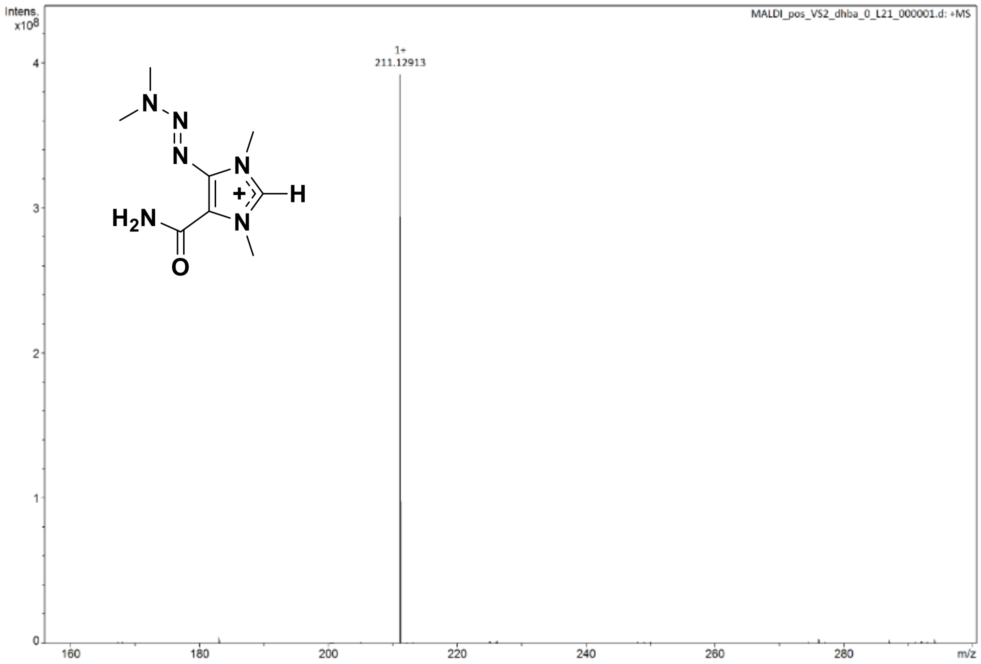

Figure S4. **MALDI-ToF (m/z):** 211.12913 attributable to the cationic portion of the imidazolium salt $[C_8H_{15}N_6O]^+$

^{1}H-NMR of the complex (NHC^d)AuCl

N, N′ dimethyl-4-[(E)-dimethylaminodiazenyl]-5-carboxamide imidazolyden gold(I) chloride

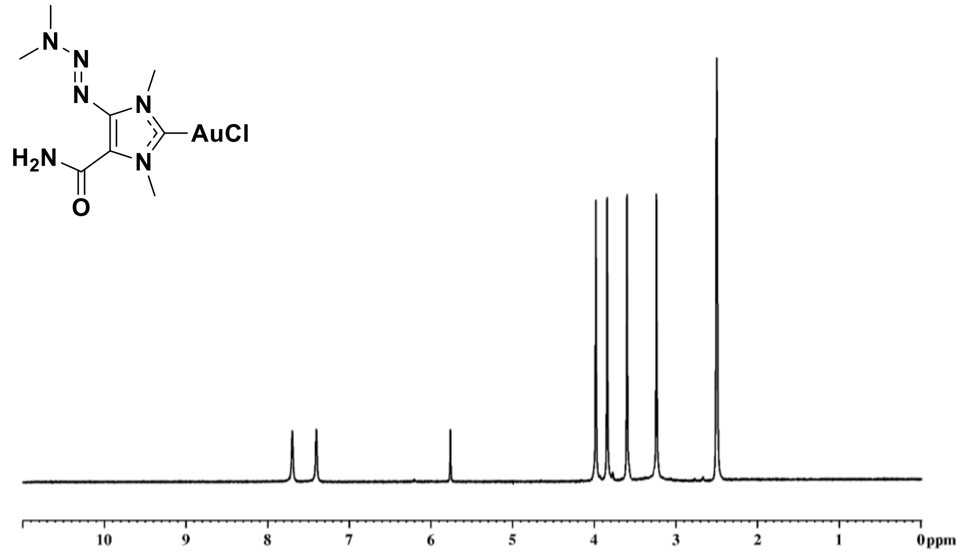

Figure S5. **^{1}H-NMR** (400 MHz, DMSO-d$_6$)ppm δ: 7.6_9, 7.4_0 (s, 2H, N***H***$_2$), 3.9_7 (s, 3H, NC***H***$_3$), 3.8_4 (s, 3H, NC***H***$_3$), 3.5_9, 3.2_3 (s, 6H, N(C***H***$_3$)$_2$)

^{13}C-NMR of the complex (NHC^d)AuCl

N, N′ dimethyl-4-[(E)-dimethylaminodiazenyl]-5-carboxamide imidazolyden gold(I) chloride

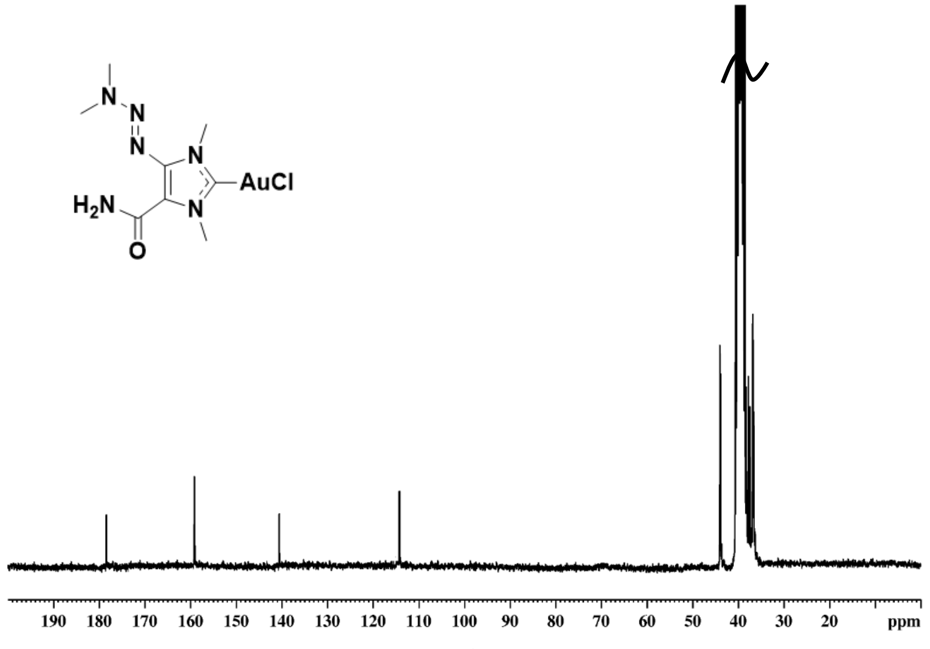

Figure S6. **^{13}C-NMR** (100 MHz, DMSO-d$_6$) ppm δ: 178.4_3 (N***C***AuN), 159.1_4 (***C***ONH$_2$), 140.7_1 (C=***C***(N=N-N)), 114.2_5 (C=***C***(CONH$_2$), 43.9_6 (NC***H***$_3$), 38.2_0 (NC***H***$_3$), 37.7_3, 37.4_2 (N=N-N(**C**H$_3$)$_2$)

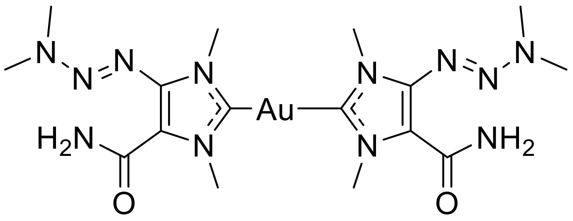

MALDI-ToF

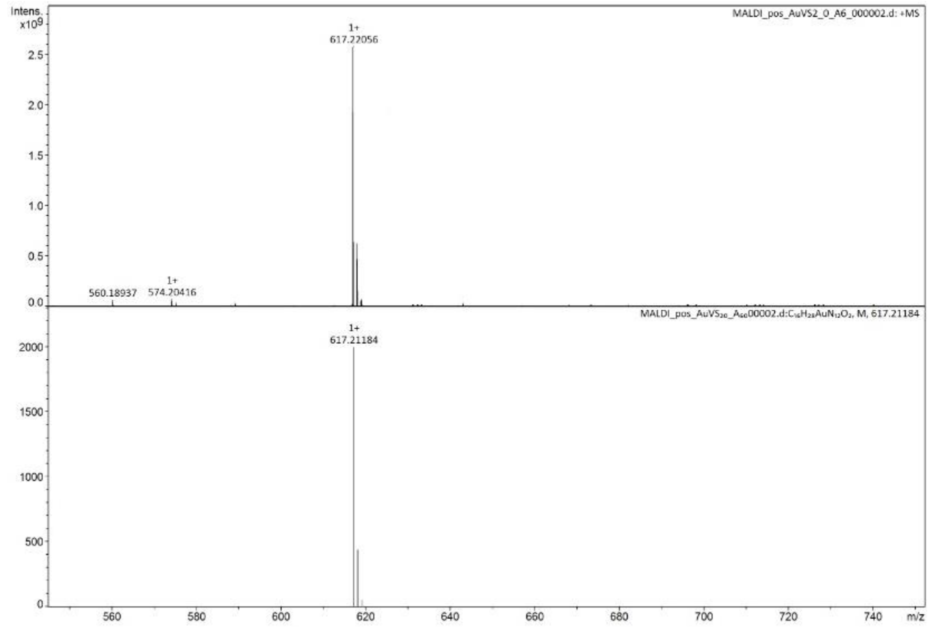

Figure S7. **MALDI-ToF (m/z):** 617.22056 attributable to a bis-carbene structure $[C_{16}H_{28}AuN_{12}O_{2}]^{+}$
